# Characterization of Shiga toxin-producing *Escherichia coli* (STEC) in feces of healthy and diarrheic calves in Urmia region, Iran

**Published:** 2012-06

**Authors:** Saei H Dastmalchi, N Ayremlou

**Affiliations:** 1 Department of Microbiology, Faculty of Veterinary Medicine and Department of Cellular and Molecular Biotechnology, Institute of Biotechnology, Urmia University, P.O. Box 1177, Urmia, Iran; 2 Graduated from Faculty of Veterinary Medicine, Urmia University, P.O. Box 1177, Urmia, Iran

**Keywords:** Shiga toxin, *E. coli*, calves, Iran

## Abstract

**Background and Objectives:**

Shiga toxin-producing *Escherichia coli* (STEC) have emerged as human pathogens and contamination of foods of animal origin has been a major public health concern. The aim of the present study was to determine the dissemination of STEC in healthy and diarrheic calves in Urmia region which is located in West Azerbaijan province, Iran.

**Materials and Methods:**

In the current study, a total of 124 *Escherichia coli* isolates from clinically healthy (n = 73) and diarrheic calves (51) belonging to 6 different farms located in West Azerbaijan province, Iran, were screened by the polymerase chain reaction (PCR) assay for the presence of virulence genes characteristic for STEC, that is, Shiga-toxin producing gene(s) (*stx1*, *stx2*), intimin (*eaeA*) and enterohemolysin (*hlyA*).

**Results:**

STEC isolates were recovered from 21.92% (16/73) in healthy calves, and 19.6% (10/51) in diarrheic calves. Overall, PCR results showed that 6 (23.1%) isolates carried *stx1* gene, 7 (26.92%) possessed *stx2* gene while 13 isolates (50%) gave positive amplicon both for *stx1* and *stx2* genes. All *stx* positive isolates were assayed further to detect *eaeA* and *hlyA* sequences. Seven out of the 26 (26.92%) Shiga toxin gene positive isolates were positive for the *eaeA* gene, and 15 (57.69%) were positive for the *hlyA* gene. Both virulence genes (*eaeA* and *hlyA*) in the same isolate were observed in 5 (19.23%) of the *stx* ^+^ isolates. In total, diverse virulence gene profiles were detected, from which isolates with the genetic profile *stx1 stx2 hlyA* was the most prevalent. In addition, *eaeA* gene was more evident in isolates from diarrheic calves than in healthy calves.

**Conclusion:**

There was no significant difference in detecting STEC isolates between healthy and diarrheic calves. It seems that calves to be the reservoir of STEC within the herds and calf management may represent specific control points for reducing STEC spread within dairy units.

## INTRODUCTION

*Escherichia coli* is the most common facultative anaerobe found in the gastrointestinal tract of warm-blooded animals and humans. In contrast to pathogenic *E. coli* strains, which typically carry certain virulence gene patterns associated with specific pathotypes (1), commensal *E. coli* strains rarely contain virulence genes (2). Pathogenic strains that are characterized by their ability to produce verotoxins (also called Shiga toxins) are known as the verocytotoxigenic *E. coli* (VTEC) or Shiga toxin-producing *E. coli* (STEC). In developed countries STEC O157:H7 represents the major cause of human diseases; however, there have been increasing reports of non-O157 STEC strains associated with gastrointestinal infections (3, 4). The pathogenicity of STEC is mediated mainly through Shiga toxins 1 (which is almost identical to Stx produced by *Shigella dysenteriae*) and 2 encoded by *stx1* and *stx2* genes, respectively (5). Many STEC also produce intimin, an outer membrane surface adhesin encoded by the chromosomal *eaeA* gene with several variants located on the pathogenicity island termed the locus of enterocyte effacement (LEE) (6). Intimin is responsible for intimate attachment of STEC to the intestinal epithelial cells, causing attaching and effacing (A/E) lesions in the intestinal mucosa (7). A factor that may also affect the virulence of STEC strains is the 60-MDa plasmid borne enterohemolysin (Ehly) which is encoded by the *hlyA* gene (8). It has been suggested that entrohemolysins may enhance the effect of Shiga toxins (9) and hemolytic activity is thought to correlate with the pathogenicity of *E. coli*. Domestic ruminants, particularly cattle and sheep, have been pointed out to be the major source of *E. coli* O157:H7 and other STEC (9, 10). Many studies also showed that both healthy and diarrheic calves harbor STEC in their intestine (11, 12) and shed the bacteria for several months and in great quantities (13, 14). Control of STEC is required at the farm level and may be achieved through a reduction of horizontal transmission within cattle groups, thus decreasing STEC prevalence (15). Therefore, the characterization of intestinal *E. coli* strains with respect to diversity, virulence trait profiles, phylogenetic affiliations, and antibiotic resistance provides important information about the health status of the harboring host and the risks for development of diseases, transmission of pathogens, or resistance to antibiotic treatment (16). In Iran, shiga toxin producing *Escherichia coli* O157:H7 has been identified in raw cow milk samples (17) and STEC isolates are one of the major etiologic agents of diarrhea (18, 19). Hence, an attempt was made in the present study to determine and compare the prevalence and virulence characteristics of STEC isolates isolated from calves with diarrhea and those without diarrhea to assess their significance as STEC reservoirs in Urmia region, Iran.

## MATERIALS AND METHODS

### Samples collection and *E. coli* isolates

During a time span of March 2010 to July 2010, fecal samples were collected from the rectum of each 2-6 months old healthy and diarrheic calves on six farms of Urmia region which is located in West Azerbaijan province, Iran. The health status of each calf was evaluated by clinical examination. Healthy calves had to be free from diarrhea, whereas sick calves showed fever, anorexia, abnormal fecal constancy and/or signs of dehydration and weakness. Fecal samples were collected into sterile plastic tubes and submitted to the laboratory on ice packs in chests. One gram of feces was resuspended in 10 ml 0.85% NaCl, and MacConkey-lactose agar plates per sample were swab inoculated and streaked for isolation. After overnight incubation at 37°C, lactose-positive (rose pink) colonies were counted as *E. coli* and further streaked onto eosin methylene blue (EMB) and incubated overnight at 37°C again. Green metallic sheen colonies indicative of *E. coli* of both the STEC and the non-STEC groups were selected and subcultured on sheep blood agar plates. All isolates were subjected to standard microbiological techniques, including indole, methyl red, Voges–Proskauer and Simmons citrate and were stored at -20°C in containing glycerol until molecular tests were carried out.

### Bacterial DNA preparation for PCR

A loop-full of overnight culture of bacterial colony was suspended in 100 µl of sterile water, boiled for 10 min and the cell debris was removed by centrifugation at 13000 rpm for 2 min. The supernatant was subsequently used as a source of DNA template (20).

### Molecular characterization of *E. coli* using the polymerase chain reaction (PCR)

The biochemical results were confirmed by PCR amplification using *E. coli* species-specific primers Eco 2083 (GCT TGA CAC TGA ACA TTG AG) and Eco 2745 (GCA CTT ATC TCT TCC GCA TT) (21). The reactions were performed in 25 µl volumes comprising of 20 ng of the template DNA, 12.5 µl of 2X PCR master mix (3 mM MgCl_2_, 0.04U/µl Taq polymerase, reaction buffer, 0.4 mM of each dNTPs), 0.4 µM of each primer. The amplifications were performed in Corbett thermal cycler (Model CP2-003, Australia) at 94°C for 4 min; 35 cycles of 94°C for 45 s; 57°C for 1 min; 72°C for 2 min and a final elongation step at 72°C for 10 min. Both positive and negative control reactions were included in each PCR amplification experiment. For negative controls template DNA was replaced with sterile water. *E. coli* O157:H7 (ATCC 43895) was used as positive control. Then, 662 bp-positive bacterial samples were tested for virulence marker genes (*stx1*, *stx2*, *eaeA*, and *hlyA*) using two duplex-PCRs (22, 23).

### Detection of virulence genes using PCR

Initial screening for STEC was performed by means of a duplex-PCR using specific primers to determine the presence of *stx1* and *stx2* genes (22). Primer sequences and predicted size of the corresponding amplified product are shown in Table 1. Reaction mixture consisted of 12.5 µl of 2X PCR master mix, 0.4 µM *stx1* primers, 0.4 µM *stx2* primers, and 2µl aliquot of the supernatant of the boiled bacterial suspension as DNA template in a 25 µl reaction volume. The PCR condition adopted in this study were similar to those previously used by Osek *et al*. (23). Amplification was performed beginning with 94°C for 5 min followed by 30 cycles of 94°C for 1 min; 53°C for 1 min; 72°C for 1 min; and a final extension at 72°C for 5 min. PCR products were electrophoresed on 1.2% (w/v) agarose gel containing 5µg/ml ethidium bromide in 0.5X TBE electrophoresis buffer for approximately 90 min at 80 v. Products were visualized by a UV transilluminator and size determination was achieved using a 100 bp DNA ladder (Fermentas, Germany). Samples showing a positive PCR result for one or both *stx1* or *stx2* were then tested for the presence of *eaeA* and *hlyA* genes with the primer pairs (8, 23, 24) using duplex-PCR as described previously by Osek *et al*. (23).

**Table 1:** Primer sequences and predicted lengths of PCR amplification products.

Virulence gene	Oligonucleotide primer (5′→3′)	Amplicon size (bp)	Ref.
*stx1*	CAG TTA ATG TCG TGG CGA AGG CAC CAG ACA ATG TAA CCG CTG	348	22
*stx2*	ATC CTA TTC CCG GGA GTT TAC G GCG TCA TCG TAT ACA CAG GAG C	584	22
*eaeA*	GGG ATC GAT TAC CGT CAT TTT ATC AGC CTT AAT CTC	837	24
*hlyA*	GCA TCA TCA AGC GTA CGT TCC AAT GAG CCA AGC TGG TTA AGC T	534	8

*Statistical analysis*. Statistical analysis was performed using GraphPad Prism version 5 software. Differences between the frequency of STEC from healthy and diarrheic calves were analyzed by Fisher's exact test. P values <0.05 were considered to be statistically significant.

## RESULTS

A total of 124 *E. coli* isolates from healthy (n = 73) and diarchic (n = 51) calves were identified by conventional as well as molecular method using species-specific primers Eco2083 and Eco2745. PCR amplification revealed a band at approximately 662 bp from all isolates, indicating the presence of bacterial DNA (data not shown). Of 124 *E. coli*, a total of 26 STEC isolates (20.97%) were identified, from which 6 (23.1%) isolates carried *stx1* gene, 7 (26.92%) possessed *stx2* gene while 13 isolates (50%) gave positive amplicon for both *stx1* and *stx2* genes (Table 2). The genes *eaeA* and *hlyA* were present in 7 (26.92%) and 16 (61.54%) of the isolates, respectively.

**Table 2:** Distribution of virulence factors of *E. coli* isolates recovered from healthy and diarrheic calves

Genetic profile	No. of isolate		Total

	healthy calves	Diarrheic calves	
*stx1*	2	1	3
*stx2*	2	1	3
*stx1* and *stx2*	2	–	2
*stx1* and *hlyA*	1	–	1
*stx2* and *hlyA*	1	1	2
*stx1*, *stx2*, *eaeA*	–	2	2
*stx1*, *stx2*, *hlyA*	5	3	8
*stx1*, *eaeA*, *hlyA*	2	–	2
*stx2*, *eaeA*, *hlyA*	1	1	2
*stx1*, *stx2*, *eaeA, hlyA*	–	1	1
	16	10	26

As shown in Table 2, isolates harboring only *stx1* corresponded to 31.25% (5/16) and 10% (1/10) of the recovered STEC isolates from healthy and diarrheic calves, respectively. *stx2* was found in 25% (4/16) and 30% (3/10) of the isolates from healthy and diarrheic calves, respectively. *stx1* plus *stx2* sequences were harbored by 43.75% (7/16) and 60% (6/10) of the isolates obtained from healthy and diarrheic calves, respectively. The *eaeA* gene was also detected in 18.75% (3/16) and 40% (4/10) of STEC isolates from healthy and diarrheic calves, respectively, while the corresponding percentages for the *hlyA* gene were 62.5% (10/16) and 60% (6/10), respectively. Homogeneity in the distribution of the later gene among different virulence gene profiles was observed. The distribution of virulence genes is presented in Table 2.

**Fig. 1:**
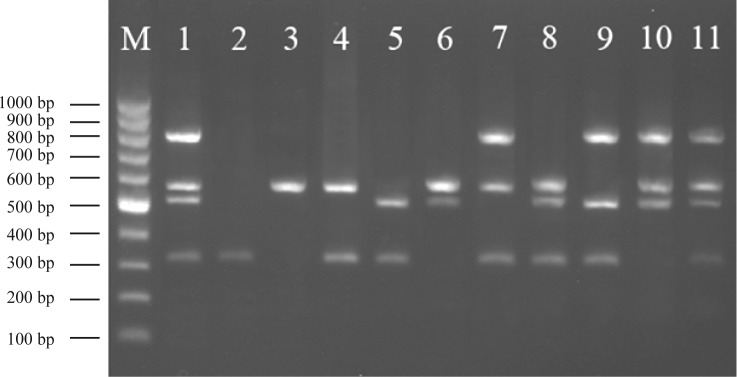
Gel for representation two duplex-PCR amplification of DNA extracted from selected *E. coli* isolates from calves showing the presence of diverse genetic profiles. Lane M: GeneRulerTM 100 bp DNA ladder marker. Lane 1: Positive control (*E. coli* O157:H7 ATCC 43895). Lane 2: profile *stx1* (three isolates); Lane 3: profile *stx2* (three isolates); Lane 4: profile *stx1 stx2* (two isolates); Lane 5: profile *stx1 hlyA* (one isolate); Lane 6: profile *stx2 hlyA* (two isolates); Lane 7: profile *stx1 stx2 eaeA* (two isolates); Lane 8: profile *stx1 stx2 hlyA* (eight isolates); Lane 9: profile *stx1 eaeA hlyA* (two isolates); Lane 10: profile *stx2 eaeA hlyA* (two isolates); Lane 11: profile *stx1 stx2 eaeA hlyA* (one isolate).

In general, STEC isolates carrying *stx1* plus *stx2* sequences were found more frequently (50%) in calves. As shown in Table 2, the virulence gene profile of the STEC isolates from calves was found in 10 diverse combinations, from which isolates with the genetic profile *stx1 stx2 hlyA* was the most prevalent. The *eaeA* gene was detected in 7 (26.92%) isolates, of these 3 isolates were positive for *stx1* plus *stx2*, 2 for *stx2* and 2 for *stx1*.

Statistical analysis showed that there was not a significant difference in the frequencies of STEC isolates between healthy and diarrheic calves.

## DISCUSSION

This study reports on the isolation of 26 STEC (20.97%) from clinically healthy and diarrheic calves in the West Azerbaijan province, Iran. Our result supported the findings of other workers, who showed that calves are possible reservoir of STEC in their gastrointestinal tract (20, 25). According to the results, 21.92% of the *E. coli* isolates recovered from clinically healthy calves carried at least one of the *stx* genes. The frequency detected in the present study is, however, lower than the 37% and 44% reported for calves in Spain and Brazil, respectively (11, 26), but higher than the 5.4% found in calves in Poland (27). These variations are likely due to geographical differences. It has been shown that he environment may have an influence on the shedding of STEC in calves (28). Individual differences in fecal excretion by cattle have also been observed, and one of the factors proposed to explain this phenomenon is climate condition (15). Higher prevalence of *stx1* gene than *stx2* gene in healthy calves, in this study, corroborates the findings of other studies (27, 29). Presence of *stx* genes-carrying *E. coli* isolates in the gastrointestinal tract of healthy calves suggests that these are transient commensal bacteria in calves and the virulence genes of these *E. coli* isolates were either not or only very poorly expressed in the intestine. This may be clarified through further research into the expression of particular genes in vivo.

With regards to isolates from diarrheic calves, 19.6% were positive for *stx* genes. The frequency detected in the present study is lower than those reported for calves in various regions (30, 31), but higher than 9.73%, 7.6% and 3.3% reported by Wani *et al*. (32), Osek *et al*. (25) and Guler *et al*. (20), respectively. Higher frequency of *stx2* gene (30%) than *stx1* gene (10%) in diarrheic calves observed in the present study is contrary to the observations of Mercado *et al*. (33) who reported predominance of *stx1* over *stx2* in diarrheic calves in Argentina. Natural outbreaks and experimental conditions have demonstrated the association of STEC with diarrhea and dysentery in calves (34, 35). However, in this study the significance of STEC in diarrheic calves is unclear because other enteric pathogens were not examined.

The relative occurrence of STEC virulence factors changed as calves aged, with *stx1*-positive isolates replaced by *stx2*-positive isolates (28). In the current study, however, *stx1* plus *stx2* sequences were present in a higher number (50%) of isolates from both healthy and diarrheic calves. This may be due to differences in age of calves, season of collection, ruminal development, immune response, diet and other aspects of calves’ management. In a study carried out by Fernandez *et al*. (36), calves in spring showed an increase of the percentage of *stx1* + *stx2* positive bacteria, indicating the fact that the relationship between age and type of *stx* is influenced by the season in which the study has been carried out.

Numerous investigators have underlined the strong association between the carriage of *eaeA* gene and the capacity of STEC to cause severe human disease, especially HUS (37, 38). In the current study, the *eaeA* gene was detected in 18.75% (3/16) and 40% (4/10) of STEC isolates from healthy and diarrheic calves, respectively. Similar prevalence of *eaeA* gene was also found in other studies (39, 40). Several authors have found that STEC isolated from diarrhoeic calves frequently share virulence-associated traits, i.e. genes with human enterohaemorrhagic *E. coli* (EHEC) strains (41).

A high frequency (57.69%) of the *hlyA* gene with uniform distribution was detected in *stx1* and/or *stx2* positive isolates. This could have resulted from the fact that it can be easily transferred among bacterial isolates since it is plasmid encoded (42). No differences of carriage of STEC in diarrheic and healthy calves were observed in this study. This is in agreement with study reported by Irino *et al*. (43) in dairy cattle (STEC prevalence among diarrheic and healthy cattle: 21.4% versus 26.4%, respectively) and is in contrast to the results previously described for beef cattle by Leomil *et al*. (44) (the frequency of carriage of STEC was higher in diarrheic calves than in non-diarrheic animals, 20% versus 8% respectively, P < 0.001) in Sao Paulo. The occurrence of virulence factors therefore does not serve as proof of the pathogenicity of a given clone. The verification of virulent *E. coli* clones as agents of disease and the distinction between virulent and commensal *E. coli* clones will require a number of different approaches, including determination of the relative proportion of a given clone to the total *E. coli*.

In conclusion, this study revealed no significant difference between healthy and diarrheic calves for carrying STEC in gastrointestinal tract, which highlight the importance of calves as a reservoir of STEC isolates in Urmia region. More serious assessment of these zoonotic pathogens in feces of calves and other animals must be carried out to determine the STEC reservoirs in this area.
